# Towards a Soil Remediation Strategy Using Biochar: Effects on Soil Chemical Properties and Bioavailability of Potentially Toxic Elements

**DOI:** 10.3390/toxics9080184

**Published:** 2021-08-04

**Authors:** Fotis Bilias, Thomai Nikoli, Dimitrios Kalderis, Dionisios Gasparatos

**Affiliations:** 1 Soil Science Laboratory, Soil Science and Agricultural Engineering, Aristotle University of Thessaloniki, 54124 Thessaloniki, Greece; fbilias@agro.auth.gr; 2 Laboratory of Soil Science and Plant Diagnostics, Mediterranean Agronomic Institute of Chania, 73100 Chania, Greece; thomainikoli@maich.gr; 3 Department of Electronic Engineering, Hellenic Mediterranean University, 73133 Chania, Greece; kalderis@hmu.gr; 4 Laboratory of Soil Science and Agricultural Chemistry, Agricultural University of Athens, 11855 Athens, Greece

**Keywords:** biochar, potentially toxic elements (PTEs), scientometric analysis, CiteSpace, soil contamination, immobilization mechanisms, designer biochars, soil-biochar interractions

## Abstract

Soil contamination with potentially toxic elements (PTEs) is considered one of the most severe environmental threats, while among remediation strategies, research on the application of soil amendments has received important consideration. This review highlights the effects of biochar application on soil properties and the bioavailability of potentially toxic elements describing research areas of intense current and emerging activity. Using a visual scientometric analysis, our study shows that between 2019 and 2020, research sub-fields like earthworm activities and responses, greenhouse gass emissions, and low molecular weight organic acids have gained most of the attention when biochar was investigated for soil remediation purposes. Moreover, biomasses like rice straw, sewage sludge, and sawdust were found to be the most commonly used feedstocks for biochar production. The effect of biochar on soil chemistry and different mechanisms responsible for PTEs’ immobilization with biochar, are also briefly reported. Special attention is also given to specific PTEs most commonly found at contaminated soils, including Cu, Zn, Ni, Cr, Pb, Cd, and As, and therefore are more extensively revised in this paper. This review also addresses some of the issues in developing innovative methodologies for engineered biochars, introduced alongside some suggestions which intend to form a more focused soil remediation strategy.

## 1. Introduction

Contamination of soils with potentially toxic elements (PTEs) is among the most serious threats to natural and environmental resources globally. PTEs are now used instead of the common term “heavy metals” as a more inclusive and appropriate term to encompass all metals, metalloids, non-metals, and other inorganic elements in the soil–plant–animal system with the potential for toxicity depending on their concentration [1,2] (Table 1). Although PTEs origin in soils may be either lithogenic (naturally in the soil environment as the result of the weathering of parent material) or anthropogenic, in most cases high PTEs’ concentrations are associated with human activities [3]. Anthropogenic sources as atmospheric deposition (coal and gasoline combustion, metal mining, smelting, industrial activities, dust from tire wear) land application of organic wastes (sewage sludge, livestock manure), the use of agrochemicals (fertilizers, fungicides), and indiscriminate land disposal of industrial waste are the main routes of PTE inputs into soils [4,5]. Moreover, because they are non-biodegradable, PTEs can, over a long time-period, be transferred from the pedosphere to the hydrosphere and biosphere representing a significant hazard to food safety, ecosystems, and human health. For example, the exposure of PTEs, particularly that of Cd, As, Pb, and Cr even when present in relatively low concentrations, may cause a series of chronic effects on nervous and immune systems, and serious diseases such as lung cancer, kidney and liver dysfunction, and bone fractures [6]. The detrimental effects are more severe in children, who are more vulnerable than adults concerning risk assessment indices [7]. As a result of the growing awareness of these problems, PTEs in soils have received widespread scientific, legislative, and public attention over the last few decades. According to Liu et al. [8], there are over 20 million ha of land contaminated by PTEs around the world leading to the deaths of almost 12.6 million people from diseases caused by contaminated soils. For the EU-28, PTEs make up around 35% and 31% of total soil and groundwater contamination, respectively [1].

Consequently, many in situ (solidification/stabilization, immobilization, soil flushing, phytoremediation, and biological/microbial treatments) and ex situ (vitrification land filling, bioreactors) soil remediation techniques have been used in the urgency of developing concrete strategies to address the causes and impacts of PTEs’ contamination. These remediation techniques include various working principles and specific advantages and drawbacks [9]. 

Among in situ methods, immobilization of PTEs with application of soil amendments is a very promising technique because of its simplicity, high effectiveness, and its commercial viability. Appropriate immobilizing agents should be abundant and applicable to use as cost effective soil amendments in order to satisfy “green and sustainable remediation” principles [10]. 

The extensive application of biochar, an organic waste-derived soil amendment, has received significant consideration in the past few years to remediate PTE-contaminated soils [11,12]. Biochar has been used to improve soil quality for millennia and can immobilize PTEs in contaminated soils by reducing crop uptake of PTEs [13]. However, the effect of biochar on PTEs availability is dependent on the type of biochar, the soil conditions, and PTE species [9]. The aims of this review are to (a) summarize recent advances in biochar characteristics focusing on production and modification methods, (b) highlight the effect of biochar in soil chemical properties that govern PTEs availability (c) better understand the complex mechanisms that control direct and indirect interactions between biochar and PTEs (d) determine the current state of knowledge of biochar application on the most commonly found PTEs in contaminated soils under specific location factors (e) unveil the knowledge gaps needing urgent attention with several research directions of novel approaches which may increase the efficiency of biochar use in soil remediation.

Thus, this review will demonstrate that there is a need to gain a better understanding of, and perform an assessment of, the suitable types of biochar for soil remediation as well as its complex interaction with specific PTEs to ensure its effectiveness for soil restoration and public health protection. 

## 2. Biochar Characteristics 

### 2.1. Introduction and Scientometric Analysis 

**Figure 1:**
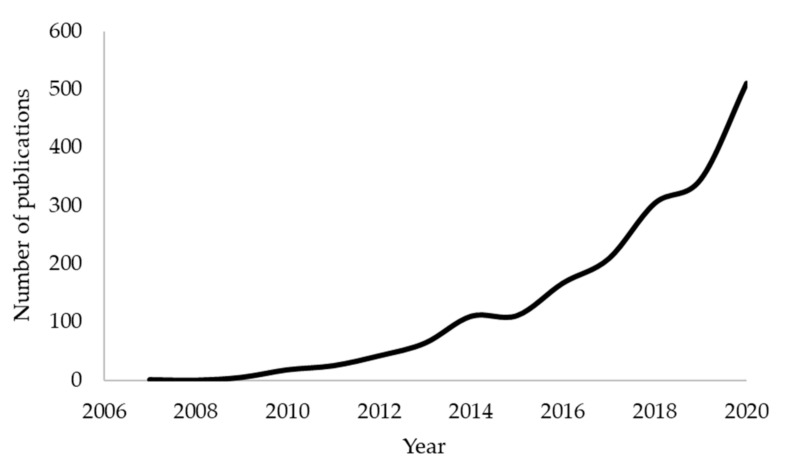
Yearly development and increasing interest in biochar research, based on the keywords ‘biochar’ and ‘soil pollution’.

Biochar is the solid, carbonaceous material produced during the thermochemical conversion (pyrolysis) of biomass in the absence of, or in limited oxygen and at temperatures in the range of 300–800 °C. Although no official definition exists, the use of the term biochar in over 8000 published works and reports, which describe more than 30 distinctly different applications, indicate that biochar should be seen as a multi-functional and multi-purpose material and not as one exclusively connected to a specific application. Over the last 20 years, the published literature on biochar has been steadily increasing and expanding in nature. From the application of biochar as a water filtration media to its use in composite building materials, new applications are continuously being developed. Between 2000 and 2006, there were 13 publications on biochar, investigating the use of the material as a precursor for activated carbon and as a soil amendment. In 2019 alone, there were 3030 published works, dealing with over 30 applications. A Scopus literature search for the terms ‘biochar’ and ‘soil pollution’ resulted in a total of 1134 papers for the period 2017–2020. A clear, increasing interest can be illustrated by the steadily growing number of publications: 205 (2017), 304 (2018), 341 (2019) and 284 so far in 2020 (Figure 1). A search within the above total for ‘heavy metals’ yielded 866 results, indicating perhaps the frequent occurrence of heavy metals among other contaminants in soil and surely highlighting the positive results obtained so far through the application of biochar. Since the term ‘potentially toxic elements’ has only recently emerged in the literature, the term ‘heavy metals’ was used to obtain more results from the search.

To locate research sub-fields of intense current activity and identify potential future trends, a visual scientometric analysis using the Java-based software CiteSpace was performed. CiteSpace was developed by Chaomei Chen in 2006 and it focuses on finding critical points in the development of a field or a domain, including identifying fast-growth topical areas, finding citation hotspots in the land of publications, decomposing a network (of publications, or authors, or geographical areas etc.) into clusters and automatically labeling clusters with the most frequent terms from citing articles [14]. The effectiveness of this approach has been shown in different fields, for example in climate change and tourism [15], trends analysis of macrophage polarization research [16] and recently in emerging trends of biochar research and applications [17].

For this purpose, Scopus was selected as the database to collect data and the search time was limited to 2019 and 2020. The keywords were ‘biochar’ and ‘soil pollution’ (article title, abstract, keywords). ‘Soil pollution’ was selected over ‘soil contamination’ or ‘soil remediation’ because it is a more inclusive phrase and resulted in a much higher number of results.

**Figure 2:**
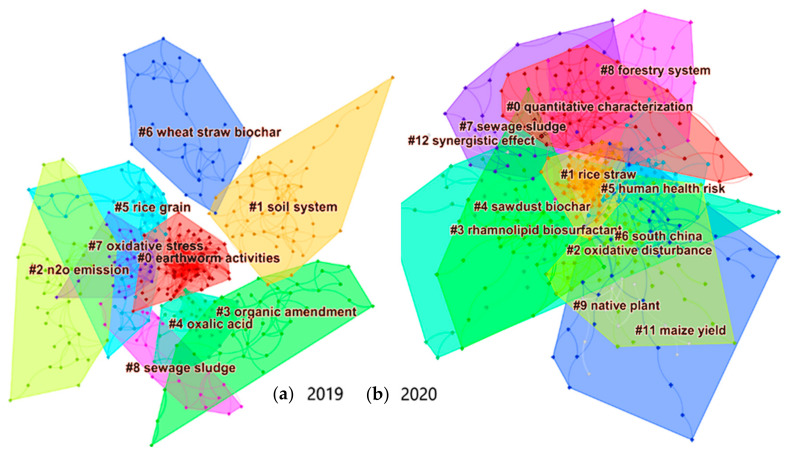
CiteSpace maps, showing the most active (most published papers) sub-fields (clusters) under the Scopus search terms ‘biochar & soil pollution’, for the years: (**a**) 2019 (341 papers); (**b**) 2020 (284 papers so far).

As shown in Figure 2, several areas (clusters) of intense activity were identified in each year. Cluster labels are selected from noun phrases and index terms of citing articles of each cluster. These terms are ranked by three different algorithms, thoroughly explained in the references above. The cluster with the lowest # number corresponds to the sub-field with the highest number of published papers. Therefore, in 2019 researchers focused on earthworm activities and responses when biochar was applied in contaminated soil. Greenhouse gases emission and specifically N_2_O emission was also well investigated. An emerging field of research is highlighted by the presence of #4 oxalic acid. Low molecular weight organic acids such as this, are released from plant roots and affect the mobilization of soil-borne nutrients and trace elements in the rhizosphere. In contaminated soils where an elevated level of trace elements is encountered, the enhanced bioavailability of trace elements may cause microbial toxicity and phytotoxicity [18]. The presence of oxalic acid may reduce the capacity of biochar to adsorb cationic metals, however more evidence is required to test this hypothesis. At #5, rice was the most studied plant species in contaminated soil cases where biochar was applied. This is to be expected since, in 2019, most related publications were produced in China which is the leading country on the production of milled rice. As indicated by #6 and #8, researchers heavily utilized wheat straw and sewage sludge for their biochar production. In the former, this is indicative of the abundance of this biomass, China again being the country with the highest wheat grain production, followed closely by the European Union as a whole. In the case of sewage sludge, the presence of available P renders this feedstock a very attractive option for biochar production and soil application, provided the concentrations of PTEs are low. 

Rice straw (#1), sewage sludge (#4) and sawdust (#7) were the most commonly used biomasses for 2020. The effects on human health (#5) and overall impact on plants (#2 oxidative disturbance, #9 native plant, #11 maize yield) have been the focus so far. Especially oxidative disturbance is closely connected to #7 oxidative stress in 2019, indicating that the plant responses to biochar-amended contaminated soils through their stress hormones, is a fundamental monitoring parameter. The combined use of biochar and rhamnolipid biosurfactant (#3) for the remediation of petroleum-contaminated soil is a new and emerging strategy, however, since this review is focused on soil contaminated with heavy metals, it is beyond our scope.

Table 2 shows the modularity value for each yearly network of publications. The modularity of a network of publications measures the extent to which the publications can be decomposed to multiple components, or modules. If a network’s modularity is close to 1.00, then the network is clearly divided into thematically distinct clusters. In contrast, if its modularity is below 0.30, one would expect to see many between-cluster links. The values for 2019 and 2020 are comparable and indicate reasonably distinct but also connected research sub-fields. The silhouette value shows the homogeneity of a cluster and takes values between −1 and 1. The higher the silhouette value, the more focused and consistent the papers of this cluster are, provided the clusters in comparison have similar sizes. The silhouette value for all 2019 and 2020 clusters showed a high degree of homogeneity, a result which was further strengthened by the increased number of papers in each cluster.

### 2.2. The Role of Feedstock in Biochar Production

Efficient management of bio-degradable waste fractions, such as forestry residues, animal manures and agricultural waste, has been a major problem throughout the world. Especially in developing countries, the lack of modernization in farming and in the processing of agricultural products leads to the disposal of large quantities of waste. Technical, financial, and social constraints further increase the quantities that remain unexploited. Production of biochar through pyrolysis offers a sustainable solution for such waste, with respect to volume reduction, bioenergy exploitation, and added-value materials.

In the early years of biochar-related research, the multi-functionality and potential applicability of the material was not fully realized. The main target was to prepare a biomass-based material to simulate the functions of the organic carbon abundantly found in the soils of Terra Preta of Brazil [19,20]. These high-carbon, fertile soils provided the example that until now researchers all over the world are trying to simulate by adding biochar to a wide range of soils and in different environmental conditions. At the same time, the porous nature of biochar and relatively inexpensive production technology meant that in certain applications it could successfully replace the more costly activated carbons. The criteria for selecting a suitable biomass were the moisture content (since it had already been established that generally biomasses with <15% moisture were suitable for pyrolysis without a pre-drying step), local or regional availability, and the need for developing sustainable management plans for biomasses that would otherwise end up in landfills. Expectedly, the first 39 biochar publications (years 2000–2008) were of a more exploratory nature and the effects of feedstock properties on biochar yield and composition were not studied in depth. In 2009, Novak and Buscher were among the first to investigate the effect of peanut hulls, poultry litter, pecan shells, hardwood, and switchgrass on biochar properties, and specifically water holding capacity [21]. The authors concluded that biochar made from switchgrass caused a loamy sand to have the highest moisture content and to remain wetter for a longer time period. In the same year, Lei et al. (2009) investigated the effect of corn stover particle size on biochar produced by microwave pyrolysis [22]. The effect of particle size was found to be insignificant on the yield, indicating that fine feedstock grinding required by conventional pyrolysis was not necessary for microwave pyrolysis process. 

Since then, many published works have investigated the effect of a wide range of feedstocks on biochar yield, properties, and specific applications. When selecting feedstocks, most researchers distinguished between high-low lignin/cellulose and ash contents. Therefore, herbaceous, woody biomasses and biosolids (such as sewage sludge and poultry litter) are often used as representatives of a wide range of lignin/cellulose and ash contents. Due to these differences, the response of each of these classes of biomass to thermochemical treatment varies. Plant-based biomass undergoes dehydration and depolymerization into smaller dissociation products of lignin and cellulose, whereas biochars derived from biosolids do not undergo depolymerisation due to the absence of a lignocellulosic fraction [23].

With respect to specific biochar properties, the effects of feedstock on pH [24,25,26,27,28], surface functional groups [26,27,29,30,31,32], cation exchange capacity [23,33,34,35], elemental analysis [29,30,36,37], ash content [26,34,36,38], and ecotoxicity [39] have been investigated. In these studies, some correlations between biomass and biochar properties were established and this enabled researchers to make progress and extend these correlations to specific biochar applications. Therefore, the influence of feedstock on the biochar’s capacity for removal of contaminants from waters and wastewaters [40,41,42,43,44,45,46,47,48], and as soil amendment [49,50,51,52,53,54,55] has been evaluated. In their recent reviews of literature, Tomczyk et al. (2020) and Vijayaraghavan (2019) thoroughly discussed the influence of biomass on biochar properties and applications, therefore a more in-depth analysis is beyond the scope of this work [23,56].

Regarding remediation of heavy metal-contaminated soils using biochar, only a few researchers have focused on the influence of the initial biomass. Gusiatin et al. (2016) prepared biochars from four distinctly different biomasses: maize silage (MS), wooden pellets (WP), sewage sludge compost (SC) and digestate residues (DR). After application to Cu-Zn-Pb contaminated soil, they concluded that SC and DR biochars very effectively reduced the mobility of Cu and Zn in the short-term (30 d), most likely due their high pH, high ash content and, in the case of SC biochar, very low dissolved organic carbon (DOC) content [26]. In another study, tomato green waste/chicken manure biochar at 5% application rate was effective in decreasing Cd bioavailability in soil and its accumulation in pak choi cultivars. Enhanced soil pH and oxygen containing surface functional groups of biochar with a microporous structure were proposed as the main mechanisms of reduction of bioavailable Cd [27]. Zheng et al. (2017) applied biochar from rice straw and maize stalk and investigated the accumulation of Cd, Zn, Pb, and As on the same cultivar. Biochars from rice straw had considerably higher pH, ash content, and cation exchange capacity (CEC) than maize stalk biochars and were more effective in immobilizing Cd, Zn, and Pb in soils, and reducing their concentrations in pak choi shoots [57]. However, maize stalk biochars were more efficient in immobilizing As. Recently, the relationship between the lignin content of the original biomass and the dissolved organic matter (DOM) leaching from biochar in an arsenic-contaminated soil, was examined. It was determined that DOM leaching from biochar decreased with increasing lignin content in the feedstock biomass and furthermore biochar from high-lignin biomass minimized the increase in As mobility [58].

All the above works have clearly shown the influence of biomass composition (lignin, cellulose, hemicellulose, and mineral content) on biochar’s physicochemical attributes. Properties such as the particle size and moisture content of biomass also play a role in the process. Generally, the following statements can be supported:

(1) The extent of mass yield loss in agricultural wastes is comparatively higher than that in woody biomass due to its higher hemicellulosic content. High yields of biochar with fixed carbon content can be achieved through pyrolysis of high-lignin feedstocks [56,59].
(2) If a biochar is expected to be applied into soil as an element supplement, a plant material containing an abundance of that element is favorable for biochar production to meet this requirement. However, metal speciation and leachability should be checked beforehand to avoid contamination issues [30,60,61]. A high inorganic content is characteristic of raw material such as the shells or husks of grains, herbaceous biomass, algal biomass, straw, and animal manure, thus leading to biochars with higher ash content. By contrast, woody materials have a lower inorganic matter content [29].
(3) Biochars from high ash biomass have larger cation exchange capacities and pH values. Therefore, they may be favorable materials for sorption of metals from soils, however, they may negatively affect nutrient availability and crop production [42,49]. Due to the higher lignin content and higher overall stability, wood feedstock biomass can be considered a better material after pyrolysis for potential carbon sequestration in soil [34,37,38,40,52,62,63]. Herbaceous biomass often leads to biochar with an increased presence of functional groups, which could serve for higher metal sorption in soil [36]. Elevated concentrations of phosphates, carbonates, sulfates, and labile organic compounds are evident in sewage sludge and manure-based biochars [60,64].

However, the lack of uniformity in the reported literature makes it difficult to correlate groups of biomasses to biochar properties, as the results are reported under different pyrolysis heating rates, reactor capacities, inert gas atmospheres, temperatures, and residence times. It is also worth noting that even a small difference (e.g., in moisture or C content) within two batches of the same biomass may result in considerably different biochars [60,65]. Finally, there are several research gaps with respect to the feedstock material for biochar and its application on metal-contaminated soil. Noticeably, data from field-scale, long-term studies investigating the interaction between biochars, multi-contaminated soils, and tree growth are completely missing.

### 2.3. Biochar Production and Modification: Towards Designer Composite Materials

Along with the biomass, the method of biochar production greatly influences its properties and subsequent applications. The fundamentals of biochar production and the influence of various reactor designs, heating rates, temperatures, and residence times have been thoroughly reviewed in the literature [59,66,67,68,69]. Furthermore, the conversion reactions for lignin, cellulose, hemicellulose, and the fate of non-lignocellulosic components have been established [70,71]. Biomass-based techno-economic analyses and several biochar-focused bio-refinery concepts have also been performed [52,72,73,74]. With respect to soil contamination, several reviews have summarized the influence of temperature, residence time, and heating rate on the efficiency of biochar to immobilize PTEs in a wide variety of soils [75,76,77,78,79]. However, little attention has been paid to biochars specifically designed and prepared through multiple experimental step, to improve their adsorption behavior in certain scenarios of PTEs-contaminated soils. Designer (or engineered) biochar gradually became a term to indicate application-oriented, outcome-based biochar modification or synthesis [80,81,82]. Therefore, the scope of this section is to evaluate innovative methodologies for engineered biochars that form part of a focused remediation strategy.

The most common approach for the preparation of an engineered material with improved performance in soil remediation is the combination of biochar and iron. Such composites synergistically combine the advantages of each of the precursors, while, at the same time, largely reduce the deficiencies of the inorganic component [83]. The deposition of zero valent iron nanoparticles (nZVI) on biochar is an efficient strategy to reduce the oxidation state of PTEs. When used alone, nZVI tends to agglomerate rapidly due to its nano-size effects and magnetic interaction, which then reduces its reactivity and mobility and decreases its remediation efficiency. Dispersed on the biochar surface, agglomeration is eliminated while the reducing power of the composite remains at a satisfactory level. This strategy has repeatedly shown positive results in Cr-, As- and Cu-contaminated soils [84,85,86,87,88,89], however field-scale trials under different environmental conditions have not yet been performed. 

Both preparation methods for nZVI/biochar, namely the wet chemistry and the thermal transformation route, have certain disadvantages. In the wet chemistry method, a Fe^2+^ donor is reduced by borohydride (BH_4_^−^) to produce nZVI, which is then attached on to the surface groups of biochar through electrostatic forces. A large-scale application would require considerable quantities of a Fe^2+^ precursor as well as borohydride. Furthermore, the properties and reactivity of the resulting nZVI particles in soil, depend mainly on the concentration, delivery rate, and volume of borohydride, reaction temperature, duration of incubation, mixing speed, solution pH, precursor concentrations and type/quantity of biochar surface functional groups [90]. Such multifactorial processes may affect the reproducibility of the final product, which in turn may generate false responses when the composite is applied in soil. The thermal transformation route involves the reduction of Fe oxides or Fe ion-impregnated biomass under reducing gases or inert gases in the presence of carbonaceous materials at high temperatures (>700 °C). Although the process is energy-demanding, it has fewer experimental variables, and more Fe can be embedded in the biochar matrix than in the wet chemistry approach.

The development of Fe-biochar composites for improved adsorption of metals through electrostatic interactions is another successful remediation strategy. In these cases, iron oxides such as γ-Fe_2_O_3_, Fe_3_O_4_ FeOS or α-FeO(OH) are precipitated on the biochar surface at appropriate concentrations and after drying, the engineered biochar is applied in soil. This approach has shown promising results in As-, Pb- and Cd-contaminated soils [91,92,93,94]. Other methodologies that have focused on developing a designer biochar with improved electrostatic attraction towards PTEs in soil, include the deposition of alginate-Fe^3+^ on biochar through one-step gelation [95], the two-stage preparation of biochar-supported iron phosphate nanoparticles composite using sodium carboxymethyl cellulose as a dispersant [96] and the three-step preparation of Ca/Fe/biochar composite [97,98]. Regardless of their efficiency, Fe-biochar composites have not been tested on the long run-in field trials, therefore questions arise with respect to the large-scale economic feasibility of the proposed methodologies. In some of these methods, strong inorganic bases (KOH/NaOH) are used to enhance iron oxide precipitation on the biochar surface. This may lead to highly alkaline engineered biochars and, as a result, adjustments to the rate or frequency of application in soil may be required. Furthermore, there is always the risk of Fe accumulation in the soil after extended use which may lead to plant toxicity. For this reason, Fe leaching tests should always be performed.

An emerging biochar engineering strategy is the single-stage production of hydroxyapatite/biochar composites, a combination known to stabilize many kinds of divalent heavy metal ions in water. Lately, it has also proved successful in the immobilization of Pb and Cd in soil, whereas at the same time promoted plant growth due to the controlled release of P [99,100,101]. At large scale, this approach would be largely affected by the cost of hydroxyapatite.

As manganese oxide and manganese oxide-modified materials have shown strong ability to immobilize As and have high oxidation potential, they can be used as potential cost-effective adsorbents for the remediation of As in soils. Yu et al. (2015, 2017) used KMnO_4_ as a precursor and deposited MnO_2_ on the biochar surface through impregnation. Then, they performed a double pyrolysis stage at 600 °C for 30 min to produce the composite [102,103]. A potential drawback of their approach is that the residual KMnO_4_ solutions they used required suitable disposal or a re-use methodology. 

Promising results have also been shown by biochar loaded with *Bacillus* sp. W1/W2 and *Microbacterium* sp. Y2, in an effort to adsorb Cr and at the same time restore the enzymatic activity and microbial diversity of the contaminated soil [104]. Similarly, biochar was used as refuge for Gram-negative bacterium *Delftia* sp. B9 and the composite was applied to the remediation of Cd-contaminated soil [105]. The main drawback of the process is that some preparation steps require sterilized conditions, a requirement not readily applicable at a large-scale. Additionally, the bacterial consortium may be sensitive to the levels of pre-existing metals and organics in biochar, therefore not all biochars are suitable as carriers. Recently, thiol-modified biochar exhibited positive adsorption behavior towards Cd and Pb in a contaminated soil. Although the preparation methodology is relatively simple, the composite has not been tested at field-scale and the mechanisms of thiol groups interaction with other metal ions are still unknown [106].

## 3. Effect of Biochar on Soil Chemical Properties

**Figure 3:**
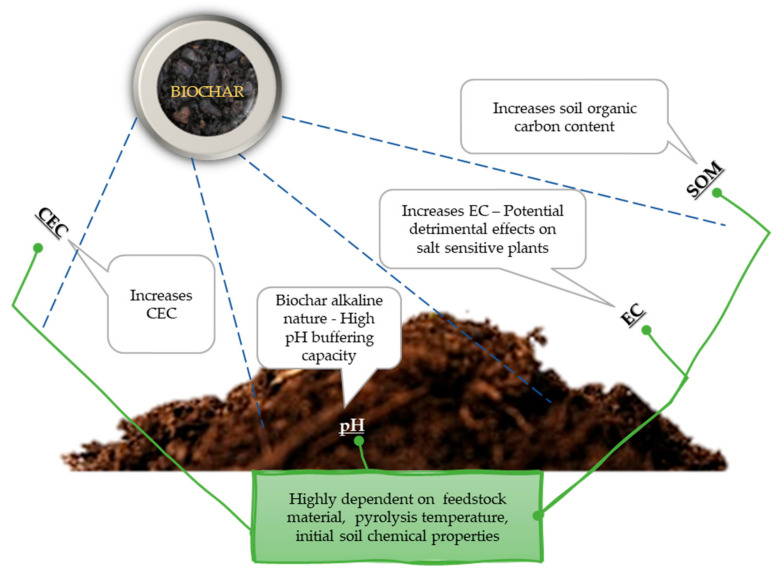
Potential effects of biochar application on basic soil chemical properties. CEC: cation exchange capacity; EC: electrical conductivity; SOM: soil organic matter.

Soil chemical properties such as pH, electrical conductivity (EC), cation exchange capacity (CEC), and soil organic matter (SOM) content, are highly impacted by the addition of biochar (Figure 3). Changes in soil chemical properties following biochar amendment can occur, however factors such as the type of soil and its initial characteristics, application rate, feedstock type, preparation conditions, and the production process of the biochar severely differentiate the extent of this impact [107,108].

### 3.1. pH

Biochars have specific properties that correct soil acidity due to their alkaline nature and high pH buffering capacity. Numerous studies have shown biochar’s role on the amelioration of acidic soils, however, in the case of alkaline soils, biochar has no positive effects on soil pH increases, and may even decrease soil pH [109]. Except for the improvement of soil’s physical-chemical properties, biochar addition has been extensively studied for the remediation of contaminated soils, since pH increase leads to reduced availability of metals (Zn, Cd, Pb, Ni) [110,111,112,113].

Peng et al. (2011) conducted a pot experiment, using biochars produced at a range of temperatures and durations, in order to examine the changes on the soil properties of an Ultisol (pH 4.7) [114]. Specifically, rice straw-derived biochars were charred at temperatures from 250 to 450 °C for between 2 and 8 h, and application rate was 1% (2.4 ton ha^−1^) for all biochars. Biochar addition resulted in a significant soil pH increase by 0.1–0.46. Response to biochar amendment varied with charring temperature and duration, whilst temperature had the most important effect. In the same line, Jien and Wang (2013) evaluated the influence of biochar made from the waste wood of white lead trees (*Leucaena leucocephala* (Lam.) de Wit) on the physicochemical of a long-term cultivated acidic Ultisol (pH = 3.95) [115]. Biochar was applied at three rates (0%, 2.5%, and 5% *wt*/*wt*) and the incubation time was 105 d for all cases. The results of their experiment showed significant increases in soil pH, especially at the highest rate of biochar.

Lu et al. (2014) conducted a pot experiment applying bamboo and rice straw biochar, on a moderately acidic (pH = 5.7) sandy loam paddy soil [110]. Both types of biochars, were pyrolyzed at temperatures ≥ 500 °C and with two mesh sizes (<0.25 mm and <1 mm), were applied at three rates (0, 1% and 5%, *w*/*w*). Soils amended with biochar had pH values significantly higher, this effect being more accentuated at the high biochar dose and small particle size. These findings were confirmed with a later study when Yang et al. (2016) concluded that soil pH increased with increasing addition rate of rice straw biochar, and the fine rice straw biochar was more effective at increasing soil pH than the coarse one [111]. However, application of bamboo biochar made no significant difference in the soil pH compared with control. The researchers attribute this fact on the higher pH, ash content, and surface alkalinity of the rice straw than the respective characteristics of the bamboo biochar.

In the field experiment of Agegnehu et al. (2016), a comparison was made among different organic amendments and several rates of inorganic fertilizer application to Nitisols [116]. The researchers evaluated the effect of biochar on improving soil properties of two sites (Holetta, pH 4.97 and Robgebeya, pH 4.83) cultivated with barley. The treatments were factorial combinations of no organic amendment (control), biochar only (10 t ha^−1^ B), compost only (10 t ha^−1^ Com), Compost + Biochar (10 t ha^−1^ Com + 2 t B ha^−1^), and co-composted biochar-compost (10 t ha^−1^ COMBI) as main plots, and five N fertilizer levels (0, 23, 46, 69 and 92 kg ha^−1^) as sub-plots. According to their results, soil pH was significantly improved by organic amendments at both sites. Addition of B, Com, Com + B and COMBI contributed to a soil pH increase by 0.51, 0.24, 0.29 and 0.27 units at Holetta and 0.52, 0.35, 0.41 and 0.48 units at Robgebeya, respectively, indicating a substantial saving on inorganic fertilizers required for the sustainable production of barley.

### 3.2. Electrical Conductivity (EC)

Similar to pH, the EC of biochar depends on the feedstock and the pyrolysis temperature. It has been proven that higher EC values occur in biochars produced at higher pyrolysis temperatures, as well as in those deriving from wood and paper waste. It is important to understand the amount of soluble salts in a biochar solution, since high rates of biochar application to soil may have detrimental effects on salt sensitive plants [117].

An increase to the soil’s electrical conductivity due to biochar addition has been reported in several studies [118,119]. Hossain et al. (2010) conducted a pot experiment with cherry tomatoes using a low initial EC chromosol (0.09 dS m^−1^) [120]. Addition of wastewater sludge biochar (pyrolysis temperature at 550 °C) at the rate of 10 t ha^−1^ was examined, alone or combined with inorganic fertilization. Application of biochar resulted in a significant increase of the soil electrical conductivity, especially in the SBF (soil with Biochar and Fertilizer) treatment, however this did not affect plant growth negatively. Sewage sludge derived biochar was also used by Méndez et al. (2012) (pyrolysis temperature at 500 °C) in order to evaluate the effect of its addition to Mediterranean agricultural soil (Haplic Cambisol, initial pH 8.63, EC 71.6 μS cm^−1^) [121]. Biochar was applied at two different rates in mass, 4% and 8%, in comparison to application of sewage sludge as the original material. The results of the experiment showed that the EC was significantly increased by the addition of biochar at both rates of application (293 and 304 μS cm^−1^, for 4 and 8% application rate, respectively), compared to the control. However, the application of sewage sludge as an original material had a bigger effect on soil EC (978 and 1124 μS cm^−1^ for 4% and 8% application rate, respectively), which indicates that pyrolysis can be used as a process for sustainable sewage sludge disposal. In the pot experiment of Luo et al. (2016), a co-composted biochar derived from peanut shells (pyrolyzed at 350 °C) was incubated for 30 days with coastal degraded soil, at four rates (0, 1.5, 5 and 10%). The impact on the growth of two halophytes (sesbania and seashore mallow) was examined. As the authors report, biochar addition led to a sharp soil salinity increase for all rates, which was even responsible for the inhibition of both the halophyte growths.

### 3.3. Cation Exchange Capacity (CEC)

Biochar can play a significant role in the amelioration of low fertility soils, through the increase of the CEC. This beneficial effect has been stated by the study of Jiang et al., 2012 where a remarkable increase on the CEC of an acidic Ultisol was shown, after incubation with 3% and 5% biochar [122]. Specifically, the CEC value increased from 3.55 for the control to 4.90 and 6.12 cmol kg^−1^ for the 3% and 5% biochar amended treatments, respectively. Moreover, Jien and Wang, (2013) examined biochar addition on highly weathered soils and reported that the CEC increased significantly from 7.41 cmol kg^−1^ in biochar-unamended soil to 9.26 cmol kg^−1^ in biochar-amended soils (application rate 2.5%), and 10.8 cmol kg^−1^ (application rate 5%), while the incubation time was also a factor that increased CEC of the amended soils [115]. Ghorbani et al. (2019) conducted a research in order to evaluate the effects of biochar deriving from rice husks (pyrolyzed at 500 °C) when applied to two soil types (loamy sand and clay), at three levels (0, 1% and 3%) [123]. According to the results of their study, the CEC of the loamy sand soil increased significantly by 20% and 30% due to the addition of biochar at 1% and 3%, respectively, while the respective increases for the clay soil were 9% and 19%. Luo et al. (2016) used a biochar-compost amendment at three rates (1.5, 5 and, 10%) in order to improve the properties and productivity of a degraded soil [119]. They reported an increase in CEC by all the application rates (up to 17.3%) compared to the control (2.72 cmol kg^−1^), as CEC was 3.03 cmol kg^−1^ at the 1.5%, and 3.19 cmol kg^−1^ at the 5%, and 3.15 cmol kg^−1^ at the 10% rate of biochar addition.

Several other studies have confirmed an increase in CEC by the addition of biochars [114,116,118,124,125,126]. However, in the literature there have also been reported cases where biochar addition had no effect on soil CEC [121,127].

### 3.4. Soil Organic Matter (SOM)

Biochar is a C-rich solid material obtained through pyrolysis of biomass. As an organic carbonaceous material, the addition of biochar to soils will inherently increase the OC content. Therefore, the application of biochar as an amendment to low fertility soils can be particularly effective in increasing SOC content, ameliorating soil quality, and mitigating climate change [108,119,128].

The increase to the soil organic carbon content caused by the addition of biochar has been reported by several authors, either referred to as the total C percentage in soil or the organic matter content [116,127,129,130]. Ajayi and Horn (2016) studied the effect of different rates of a wood chips biochar (pyrolyzed at 500–600 °C) amendment on the properties of two soils (fine-sand and sandy loamy silt), by adding 20, 50 and 100 g biochar kg^−1^ (by dry weight) [131]. The result showed that biochar amendment improved total carbon content of both soils, and based on previous studies, the authors suggest that this can be attributed to either the contribution of organic carbon by the biochar itself, or the sorption of organic matter and nutrients by the added biochar, which increased the contents of water-extractable organic carbon. Ippolito et al. (2016) in their pot experiment reported an increase of soil organic carbon, up to six times greater than the control, when they added an acidic biochar derived from mature switchgrass (pyrolyzed at 350 °C), to a calcareous eroded soil. Application rates were 0, 1, 2, and 10% (wt) and the impact on the SOC content followed a proportional trend [132].

Kizito et al. (2019) conducted a study using two types of biochar, corn cobs (CB) and wood obtained from pruning of fig trees (WB), produced at slow pyrolysis at 600 °C. Biochars enriched and unenriched with anaerobic digestate nutrients were added to a low fertility clay loam soil, combined with inorganic fertilizers. The results of this study showed that the addition of biochar alone or in combination with digestate nutrients/NPK significantly increased the SOC over the control and unamended NPK treatments [126]. Specifically, the addition of digestate-enriched biochar increased SOC by 231.9% and 370% in CB and WB treatments, respectively, compared to 224.8% and 188% resulting from unenriched WB and CB application, respectively. The authors report that since the amount of carbon added to the soil for was equal for both biochars, it is the rate of carbon mineralization that differentiated the SOC among the different types of biochar, and as reported by previous studies, the increase in SOC for digestate-enriched biochar treatments could be attributed to the sorption of liable organic matter in the digestate that is released into the soil.

## 4. Effect of Biochar on Potentially Toxic Elements: Immobilization Mechanisms

**Figure 4:**
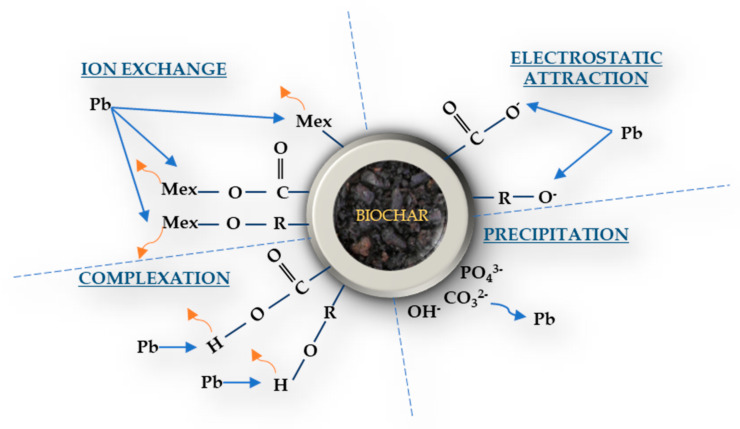
Potential mechanisms of biochar interactions with cationic PTEs in soil. The example of lead (Pb), Mex: exchangeable ions (Mg^2+^, Ca^2+^ etc.) (modified from [134]).

Pore distribution of biochar materials can exhibit large pore sizes, an extensive network of micropores and cracks, as well as pore volumes with high CO_2_ measured surface areas, which in many cases may be greater than 100 m^2^ g^−1^ [67]. The above properties, significantly related to the pyrolysis preparation procedure [133], may equip biochar with high sorption behavior, which in turn renders it as a potential sorbent for PTEs contaminants in soils [78]. However, different immobilization mechanisms under which the bioavailability of PTEs in contaminated soils can be reduced by biochar amendment, is rather difficult to define uniformly, since different biochar characteristics, and consequently the different effects that may cause on soil chemistry, (discussed in detail in Section 2 and Section 3), might occasionally activate different mechanisms, or a set of mechanisms, which in turn can affect in different ways a specific, or group, of PTEs [12]. Thus, factors such as diverse sources of raw materials, specific pyrolysis temperatures under which biochar is produced, possible modifications on biochar preparation, as well as biochar’s physical and chemical properties such as pH, EC, CEC, or organic carbon content, to a large extent indirectly determine the behavior of potential biochar–PTEs interactions. Nevertheless, the above possible mechanisms of direct interactions between PTEs and biochar (Figure 4) could be grouped into four distinct categories (electrostatic attraction, ion exchange, complexation, precipitation), which are briefly discussed below.

### 4.1. Electrostatic Attraction

Electrostatic interactions can occur between biochar’s surface charge and metal ions leading to the reduction of their bioavailability in contaminated soils. When produced at high pyrolysis temperatures, biochar’s surface is negatively charged, also promoting the formation of graphene structures favoring electrostatic attractions sorption mechanisms [135], attracting positively charged ions, and generating their immobilization. The latter is highly dependent on the soil, or biochar pH, as it changes the metal speciation in the soil matrix [136]. In two recent studies conducted for investigating biochar’s ability to immobilize Pb and Cu from shooting-range contaminated soils [137,138], electrostatic interactions found as one of the main immobilization mechanisms generated after the amendment, whereas notably, the above was reported even at low pyrolysis temperatures (300 °C) preparation [138]. Nevertheless, electrostatic attractions may also occur between negative charged ions bound on positively charged biochar’s surfaces. For example, Dong et al. (2011), studying the effects of biochar on hexavalent chromium removal from aqueous solutions, reported that on highly acidic conditions, negatively charged hexavalent Cr, was effectively bound with biochar’s surface derived from low pyrolysis preparation (300 °C) [139].

### 4.2. Ion Exchange

Cation exchange is considered as one of the main mechanisms responsible for PTEs attraction to biochars surfaces, due to the high levels of CEC that they generally have [66,137,140]. Thus, inorganic mineral cations that are in abundance on biochars surface like Ca^2+^, Mg^2+^, Na^+^ or K^+^, could be exchanged with contaminant cations which are highly concentrated in the soil solution phase of contaminated soils. The above reactions take place rapidly, are reversible and stoichiometrically defined, while they are highly dependent from both charge density of the respective cations (ions of higher charge density are preferably sorbed), as well as from their initial concentrations on soil solution [141]. Feedstock material that is to be used for biochar preparation, as well as pyrolysis temperature conditions, are major parameters in determining the effect of ion exchange reactions on contaminant cations immobilization. For example, Lei et al. (2019), comparing two types of biochar (animal-derived, and plant-derived), reported that on animal-derived biochar, the ion exchange mechanism was found as the major immobilization mechanism for both Cd and Cu [142]. In addition, Lehmann and Joseph (2015) reported that the high ash content of biochar, derived at high pyrolysis temperatures, increases the content of cations like Ca^+2^, Mg^+2^, Na^+^ or K^+^, which in turn increases its supply efficacy for cation exchange [67].

### 4.3. Complexation

Complexation, as a binding mechanism of PTEs from contaminated soils, involves specific outer- or inner-sphere metal-ligand interactions, which in turn lead to the formation of multiatom complexes that reduce the bioavailable forms of contaminants [143]. The functional groups existing in the biochar surface that are participating to the above interactions usually contain hydroxyl, carboxyl, or carbonyl groups (-OH, -COOH, -C=O), while it has been reported that the oxygen content of the above groups increases over time due to oxidation processes, leading to an overall complexation increase [144].

Biochar’s complexation efficiency has been reported to be highly related to the feedstock material that is to be used for biochar production, as well as to the initial total content of the functional groups that are to be involved in complexation interactions. For example, Inyang et al. (2016), reviewing the biochar’s immobilization mechanisms from various studies, reported that complexation of metallic species is more likely to occur with plant-derived than animal-derived biochars [143]. In the same line, and regarding the overall presence of specific functional groups in different types of biochars, Tong et al. (2011), showed that although Pb, Cu, and Cd could be both adsorbed specifically by biochars derived from different crop-residues (peanut and canola straw), the higher content of total oxygen-containing functional groups in peanut straw biochar was responsible for its higher (via complexation mechanism) adsorption capacity [145].

### 4.4. Precipitation 

The formation of precipitates between the mineral elements contained in biochar surface and PTEs of contaminated soils, is also considered as one of biochar’s major adsorption mechanisms that leads to the overall reduction of the bioavailability of inorganic contaminants. Precipitation might occur either in solution or on surface during the sorption processes, forming insoluble solid(s), while it is reported that the above mechanism is highly related to pH, as well as with pyrolysis temperature conditions of biochar preparation. For example, Jiang et al. (2012) showed that the increase in pH values caused by biochar application in an acidic contaminated Ultisol, promoted the formation of precipitates that significantly reduced bioavailable forms of Cu and Pb [122]. In addition, and as far as the pyrolysis temperature is concerned, Boostani et al. (2020), found that biochars produced at 500 °C were significantly more effective in enhancing Ni stabilization than those produced at 300 °C, likely due to their higher ash and calcium carbonate content and lower organic matter content, which consequently promoted Ni precipitation [146]. 

Source material mineral contents that form insoluble precipitates with metallic ions, usually include sylvite (KCl), quartz (SiO_2_), amorphous silica, calcite (CaCO_3_), hydroxyapatite (Ca_10_PO_4_)_6_(OH)_2_), or anhydrous calcium sulfate (CaSO_4_), which in turn exist in either free forms or are intercalated within the carbon matrix of the biochars [143]. For instance, Pellera and Gidarakos (2015) reported precipitation as CdCO_3_ and co-precipitation with CaCO_3_, as the main mechanisms contributing to Cd sorption by olive pomace-derived biochars [147]. Respectively, and regarding Pb immobilization via precipitation, Liang et al. (2014), showed that when inorganic P is desorbed from P-rich biochars in Pb-contaminated soils, Pb is likely to precipitate as Pb_10_(PO_4_)_6_(OH)_2_ and Ca_2_Pb_8_(PO_4_)_6_(OH)_2_ [148]. In the same line, Xu et al. (2013) also suggested that minerals like P play a key role in the metal retention, probably through the formation of metal phosphate precipitates [149].

## 5. Effect of Biochar on Specific Potentially Toxic Elements Mobility and Bioavailability

### 5.1. Cu

Although copper (Cu) is an essential micronutrient for plants, elevated concentrations in soil can be toxic to most plant species (>20–30 mg Cu kg^−1^ dry weight), and consequently may have negative effects, including reducing crop yield and affecting soil biodiversity [150,151]. Mining, smelting, land applications of sewage sludge, as well as the use of fungicides containing Cu, are the main anthropogenic activities responsible for soil Cu contamination, whereas the latter raises the most concerns for agricultural soils, even for areas of organic agriculture, due to the widespread use of Cu-based fungicides, including organic based ones [152,153]. 

In general, as has been reported in literature, biochar application in Cu contaminated soils, seems to be effective in immobilizing Cu^2+^ cations, a fact which is mainly conditioned by its sorption properties and the increase that offers in the content of organic carbon and in soil pH. In addition, the fact that the latter mechanisms are respectively activated for most PTEs with cationic behavior, renders biochar application as a more comprehensive remediation strategy framework for PTEs contaminated soils. Chen et al. (2018), conducting a large-scale meta-analysis study on the effects of biochar on the availability of PTEs, containing a total of 1298 individual observations worldwide comparing control (no biochar treatments) and biochar-amended treatments. This study showed that the mean concentrations of Cu in plant tissues decreased by 25%, when the plants were grown in soils amended with biochar, while the average concentrations of available Cu, in soil were reduced by 29%, respectively [154]. However, the initial concentration of Cu on raw materials that are to be used for biochar preparation may significantly alter biochar’s immobilization efficacy, or even cause negative effects, by supplying additional quantities of available Cu^2+^ on soil interior environment [155].

As far as the biochar efficacy in controlling Cu leaching loss is concerned, Bakshi et al. (2014), conducting a column leaching experiment in contaminated sandy soils, reported that biochar application was effective in binding Cu up to 41% for an Alfisol and up to 43% for a Spodosol, showing that on sandy soils, the effects of biochar could be more drastic [156]. The same authors found that biochar application managed to reduce Cu leaching loss from ~47 to 10% for the Cu-spiked Alfisol and from 48 to 9% for the Cu-spiked Spodosol, respectively. Concerning the specific mechanisms behind this immobilization behavior, they reported that Cu was likely retained on biochar surfaces through complexation, as suggested by the Fourier-transform infrared spectra analysis they conducted. In the same line, Jones et al. (2016) using an alkaline (pH = 10) specialized biochar agent, found an even more significant decrease in leachable Cu (91%), associated with 3% *w/w* of biochar application on a Cu contaminated sandy loam soil, suggesting that under special treatment, biochar application could fairly support sunflower plant growth and biomass production [157]. 

The feedstock material that is to be used for biochar production, as well as pyrolysis temperature conditions, plays a key role in determining its effectiveness in Cu immobilization or precipitation, since they highly influence properties that control biochar’s adsorption/precipitation behavior such as pH, CEC, porous structure, available functional groups, and ash content. For instance, Gonzaga et al. (2018) reported that orange bagasse derived biochar managed to better reduce Cu availability (up to 14.2%) in an acidic sandy loam Cu contaminated soil, in comparison with coconut shell or sewage sludge derived biochar [153]. Furthermore, Jiang et al. (2015), conducting batch experiments to study the effectiveness of rice straw-derived biochar on Cu adsorption [158], categorized biochar derived from different feedstock materials in terms of their maximum Cu adsorption capacity as follows: hardwood and corn straw biochar < canola straw = rice straw < peanut and soybean straws [145,159] (Tong et al., 2011; Chen et al., 2011).

Regarding the effects of pyrolysis temperature on Cu mobility, Jiang et al. (2015) indicated that Cu adsorption capacity of biochar decreased with increasing pyrolysis temperature and that more Cu could be adsorbed on the surface of the rice straw biochar produced at lower temperatures [158]. Corroborating with this, Uchimiya et al. (2011) also found that biochars obtained from cottonseed hull showed a maximum sorption performance at low pyrolysis temperature (350 °C) and better reduced Cu concentration in soil solutions when amended to an acidic sandy loam soil, in comparison to other tested pyrolysis temperatures (500 °C, 650 °C, and 800 °C, respectively) [133]. Similarly, Wei et al. (2019) also reported that low pyrolysis temperature (350 °C) attributed a better sorption performance to Jerusalem artichoke stalks derived biochars (17.0 mg g^−1^), in comparison to other tested pyrolysis temperatures (15.2 mg g^−1^ for 700 °C, and 11.1 mg g^−1^ for 500 °C, respectively) [160]. However, contradictory results have also been reported in literature, indicating that the nature of the biomass feedstock plays a crucial role [161].

Biochar’ s efficacy in reducing Cu phytoavailability in Cu contaminated soils, has been reported in various studies and for various plant species. Gascó et al. (2019) conducting a pot experiment with *Brassica napus* in a mining soil amended with rabbit manure biochar, reported that Cu concentration was significantly reduced in both shoots (up to 82.6%) and roots (up to 38.3%), after two months cultivation [162]. The same beneficial effects have also been reported for Chinese cabbage (up to 50.4% reduction of Cu concentration in roots and shoots) from Salam et al. (2019), and for ramie (*Boehmeria nivea* L.), in which plant tissue Cu reduced correspondingly to biochar dosage increase [163]. However, high capacity of each plant species for Cu uptake may lead to opposite results. For instance, Gonzaga et al. (2018), reported that despite the reduction in soil Cu availability by biochar application, it did not manage to reduce Cu uptake by Indian mustard (*Brassica juncea* L.), probably due to mustard’s high absorption efficacy [153]. Corroborating to this, Meier et al. (2017) reported that roots of a native metallophyte (*Oenothera picensis*) accumulated more Cu when biochar was added, probably due to the complexation of Cu in soil and biochar-derived DOC [164]. 

### 5.2. Zn 

Zinc is considered an important micronutrient for cellular enzymatic functions, protein production and membrane integrity, however, at excessive concentrations it is considered phytotoxic [165]. Furthermore, high amounts of zinc may also inhibit copper absorption by plants, thus resulting in copper deficiency symptoms. Zinc occurs naturally in soil (about 70 mg kg^−1^ in crustal rocks) [166], but in many parts of the world Zn concentrations are rising unnaturally, due to anthropogenic activity. It is often found in soils contaminated from a wide range of industrial activities, such as mining, coal, waste combustion and steel processing. 

The toxicity of high Zn concentration (>25 mg kg^−1^) in soil has been well documented in various species of flora and microbial biomass [167,168,169,170]. It has also been established that it may act synergistically in the co-presence of other metals and still show phytotoxic effects in coarse- and fine-textured soils after several years [171,172]. The presence of Zn in contaminated soils is often accompanied by the presence of other metals. Therefore, remediation strategies do not usually focus on Zn removal but rather on metals that have exhibited higher eco-toxicities, such as As, Pb and Cd. 

Among the first groups that investigated the use of biochar in remediation of Zn-contaminated land was that of Beesley et al. (2010), who specifically studied the effect of biochar on the mobility and bioavailability of several PTEs in a former gasworks site with acidic soil [173]. Knowing that Zn is relatively insoluble in aqueous solutions at pH values > 7, the authors succeeded in increasing the soil’s pH by 2.1 units (from 5.45 to 7.56) by adding biochar at a 2:1 soil-to-biochar ratio. After 28 days of incubation, the concentration of Zn in the pore water of the biochar-amended soil was measured four times less compared to the pore water of the original soil. Beesley and Marmiroli (2011) confirmed the above findings in a different acidic soil contaminated with Zn (249 mg kg^−1^) [174]. The concentration of Zn in the first fraction of the eluate from the biochar-amended soil was reduced to 10 μg L^−1^, compared to 270 μg L^−1^ measured in the eluate of the non-amended soil. They concluded that in addition to the alkalinization process, irreversible sorption of Zn on the biochar porous surface significantly contributes to Zn immobilization. Houben et al. and Sneath et al. also noticed this biochar effect on a soil from a zinc smelting plant (Zn 2980 mg kg^−1^) and Sn mine spoil heap (Zn 47 mg kg^−1^), respectively [175,176]. However, they questioned biochar’s long-term metal immobilization efficiency should the soil pH decrease due to environmental conditions, a concern also expressed by others [177,178,179].

Using contaminated soil from a gasworks site, Gomez-Eyles et al. (2011) investigated the behavior of *Eisenia fetida* earthworms when biochar was added in a multi-metal contaminated soil. Biochar alone was not successful in Zn immobilization, whereas when combined with the earthworms the bioavailability of Zn increased (131.9 compared to 94.9 μg L^−1^ in the pore water after 28 days of incubation) [180]. Interestingly, this led to higher concentration of Zn in the earthworm tissues (131.5 compared to 121.6 mg kg^−1^ in the non-amended soil). The same pattern was observed from Pukalchik et al. (2018), who found that 5% wt. biochar increased Zn immobilization and resulted in high *Eisenia fetida* mortality [181]. Both of these studies indicate the need to include soil biota responses when investigating the interaction between biochar and contaminants. The study from Gomez-Eyles et al. was one of the first to propose that only biochars with a much higher CEC than soil can be an effective remediation treatment for inorganic contaminants. The results of Méndez et al. agreed with this proposal. They applied sewage sludge biochar (1250 mg kg^−1^) in a sandy-loam agricultural soil and found that the soil’s pH and CEC remained practically unaffected after the addition [121]. Zn mobility and bioavailability was even slightly increased in the biochar-amended soil, although one should take into consideration the high concentration of Zn in biochar originally, compared to Zn in the test soil (48.4 mg kg^−1^). Quite the opposite results were obtained by Gondek et al. (2016), who applied 2% wt. poultry litter biochar (Zn 358 mg kg^−1^) in a sandy soil (Zn 23.9 mg kg^−1^). The authors achieved >95% immobilization of Zn due to the large increase in the soil’s pH and CEC after biochar addition [182]. 

The earlier studies did not focus on the quantity of biochar applied but more on the effects of various biochars on a wide range of soils and establishing the mechanisms of interaction [133,177,183,184]. Subsequently, researchers investigated the effects of biochar on Zn bioavailability in combination with plant species grown in contaminated soils. Most studies solidly support that biochars can reduce Zn bioavailability and at the same time promote the growth of plants and microbial biomass. Prapagdee et al. (2014) applied low-temperature biochar at a rate of 10% which reduced the bioavailability of Zn and at the same time improved the growth rate of *Vigna radiata* L. [185]. The same trend was observed when Puga et al. (2015) added 5% wt. of high-temperature biochar in a mining soil (Zn 2960 mg kg^−1^) [178]. The accumulation of Zn in the roots and shoots of *Canavalia ensiformis* and *Mucuna aterima* was reduced by more than 50% and 30%, respectively. At the same biochar application rate, very similar reductions were achieved on *Dactylis glomerata* L., *Brassica chinensis* L., *Oryza sativa* L., *Salix viminalis* L., *Lolium perenne* L. and *Lolium multiflorum* L., leading to improved growth of the species on either acidic or alkaline soils [57,186,187,188,189,190]. By applying olive tree prunings biochar to a mining soil at a rate of 10%, Beesley et al. (2014) confirmed his earlier findings regarding Zn. However, they noted that As—which co-existed in the soil with Zn—was mobilized and lead to increased phytoxicity. This was a clear warning that in a multi-metal contaminated soil, biochar addition may affect the mobility and bioavailability of some metals in opposite ways, thus increasing the environmental threat [191]. Regarding the soil microbial biomass, it was increased from 750 to 1100 μg g^−1^ when biochar was applied in an alkaline soil at a rate of 5% wt. [192]. The concentration of Zn in pore water was reduced from 700 to 25 μg L^−1^ in the biochar-amended soil thus following the established trend of Zn immobilization by biochar. It is important to note that the levels of microbial biomass were strongly negatively correlated with the bio-available Zn.

The importance of the pre-existing Zn concentration in biochar was emphatically stated in Rodriguez-Vila et al. (2015, 2016). Although the authors applied much higher biochar doses than others (20, 40 and 80% wt.), they found that Zn concentrations in the soil mobile fractions and in the plant *Brassica juncea* L., were generally higher in the biochar-amended soils compared to the untreated soils, suggesting than Zn was added by biochar itself [193,194]. Chao et al. (2018) further elaborated on the phyto-availability of Zn by applying peanut shell biochar (1–5% wt. treatments, biochar Zn concentration 61.1 mg kg^−1^) in an acidic mining soil contaminated with 304.5 mg kg^−1^ of Zn [195]. All treatments reduced the mobility of Zn, however increased concentrations were determined in the husk and grain of *Oryza sativa* L. The authors added their concern to that of earlier workers, with respect to the long-term immobilization efficiency of biochar and the conversion of potentially contaminated feedstocks (such as sewage sludge) into biochar [196]. 

The work of Egene et al. (2018) was practically the first that investigated the long-term effect of biochar addition in a Zn-contaminated soil (298 mg kg^−1^). A single dose of 4% wt. biochar, resulted in an average decrease in Zn concentration in the pore water of 77%, over a monitoring period of 3 years [197]. The long-term results of He et al. (2019) were equally positive. When the authors applied kenaf core and sewage sludge biochar in an acidic soil at a rate of 4% wt., the immobilization capacity of biochar for Zn remained practically unchanged during a 2-year natural aging process [198]. This was due to minerals like goethite which was sorbed on biochar surface and acted as an iron-oxide source to sorb Zn.

Lately, researchers have focused on developing engineered biochars by fine-tuning the production conditions. Knowing that biochar surface area and porosity plays an important role in Zn retention in soils, Igalavithana et al. (2018) investigated the use of CO_2_ instead of N_2_ during pyrolysis. The authors produced biochars with a higher surface area and aromaticity which exhibited a higher Zn immobilization capacity compared to that of N_2_-prepared biochars [199]. In a similar engineering strategy, Hemati-Matin et al. (2020) developed nano-clay and nano-Fe biochar composites in order to improve the surface area and achieve an improved Zn retention in an alkaline soil (Zn 67.9 mg kg^−1^). At a rate of 5% wt., the composites showed an average reduction of 30% in Zn leaching from soil. It is worth noting that after the composite application, the soil pH dropped, however Zn retention was not affected. This comes in contrast with the established trend described earlier. The authors attributed their observation to the strong sorption of Zn on the composites’ surface [200].

Conclusively, the difference between the pH, CEC values of soil and biochar and the initial Zn concentration of each, plays a critical role in Zn mobility and bioavailability. Biochar application in the range of 1–5% wt. appears to be the optimum in most cases, regardless of soil type [201,202]. Having in mind an extended field application, any higher rate would perhaps be economically unfeasible and/or impractical in terms of obtaining the initial biomass for biochar production. The vast majority of data have come from short-term studies restricted to small-scale (pot) or greenhouse studies, whereas long-term studies at field-scale are largely missing.

### 5.3. Ni

Application of biochar for soil remediation purposes on Ni contaminated soils has been also a subject of various studies over the last years. Khan et al. (2013) studied the influence of sewage sludge derived biochar (pyrolyzed at 550 °C) at rates 0, 5 and 10% *w*/*w*. Rice (*Oryza sativa* L.) was planted in pots containing a serpentine (rich in Ni due to geochemical reasons) acidic soil (pH 4.02) [196]. The authors reported a decrease in the bioavailable concentrations of Ni (EDTA-extracted) after the addition of both rates of biochar to the soil tested, specifically up to 2.9–7%, attributing this fact to the increase of pH, CEC and DOC content of the amended soil. Most importantly, a subsequent significant was shown for Ni accumulation in the grain, leaves and straw of rice. Similar Ni soil conditions were investigated in the pot experiment of Herath et al. (2014), who examined the potential of biochar (a waste byproduct of bioenergy industry) as a soil amendment to immobilize Ni in serpentine soil and minimize Ni phytotoxicity for tomato (*Lycopersicon esculentum* L.) [203]. In this study, addition rates of biochar were 1, 2.5, and 5% (*w*/*w*) and in order to evaluate their effect on soil Ni fractions, the sequential extraction scheme proposed by Tessier was utilized. The results of this study showed that biochar addition, especially at the highest rate 5%, significantly decreased Ni bioavailability and potentially available concentrations (exchangeable + bound to carbonates fraction). More specifically, biochar addition led to a 14%, 36% and 61% reduction of Ni bioavailable levels, for the three rates of application, respectively, compared to the unamended soils. The authors conclude that the addition of biochar to serpentine soil can potentially immobilize Ni in serpentine soils and reduce metal-induced toxicities in tomato plants.

The potential of biochar for Ni immobilization was studied by Ehsan et al. (2014) in a soil columns experiment [204]. More specifically, biochar derived from unfertilized dates (pyrolyzed at 500 °C), was added at the rates of 0.5, 1, and 2% (*w*/*w*), on a sandy loam alkaline soil artificially polluted with 100 mg kg^−1^ Ni. The researchers studied the impact of BC addition on the water soluble, exchangeable, and total concentrations of Ni. According to their findings, the highest rate of application (2% *w*/*w*) had the most pronounced effect on the water soluble Ni content (0.29 mg kg^−1^) compared to the control, whereas the respective reduction for NH_4_NO_3_-extractable Ni was up to −53% and for the total Ni content was −50%, indicating that biochar succeeded in immobilizing Ni through the adsorption mechanism. Rees et al. (2014) observed a reduction of CaCl_2_-extractable nickel in an acidic contaminated soil (pH 5.8) after the addition of a wood derived (10% *w*/*w*) biochar in one week [177]. The researchers conclude that the observed immobilization could be due to the increase in soil pH, and the subsequent metal retention on soil particles. In contrast, for the second soil examined in this study, biochar addition did not affect Ni availability, probably because of the alkaline pH of the soil (pH 8.2).

In the study of Shen et al (2016), the long-term effect of hard wood biochar pyrolyzed at 600 °C (rates of application 0.5–2%) on the immobilization of metals in a contaminated site was investigated with a field remediation treatment [205]. Using standard leaching tests and a 5-step sequential extraction procedure, they found that the extracted nickel concentrations in the carbonic acid leaching tests were reduced by 83–98% over three years, while the residue fractions increased from 51% to 61–66% through competitive sorption, which may be responsible for the subsequent reduced leachability of Ni from 0.35% to 0.12–0.15% in the biochar treatments, compared to the control plots.

### 5.4. Cr

Chromium may exist in several oxidation states, from −2 to +6 [206]. However, in the natural environment it mainly exists in two stable states as trivalent (Cr(III)) and hexavalent chromium (Cr(VI)) [207], while considerably higher contents are found in ultrabasic extrusive igneous rocks which may reach up to 1600 mg Cr kg^−1^ [208]. The most toxic form of chromium for plants, animals and consequently for human health is in its hexavalent oxidation state due to its high solubility, mobility, and strong oxidation ability, as well as due to its ability to be easily sorbed by clays and hydrous oxides [139,209,210]. In soils, except the naturally occurring entry paths through the weathering of rocks, or volcanic eruption [208], the anthropogenic sources of Cr mainly include electroplating and sewage sludges, Cr pigment, as well as tannery and leather manufacturing wastes. 

Among different techniques that are used to reduce the concentration of toxic forms of Cr in the environment (sedimentation, chemical precipitation, membrane separation, ion exchange, ultrafiltration, and adsorption) for Cr contaminated soils, the application of biochar has gained attention in recent research studies due to its comparative low cost and its high adsorption behavior. The latter is mainly attributed to its high surface area, cation exchange capacity, and the number of its available functional groups [121,211].

In general, as has been cited from various studies, the addition of different types of plant or animal-derived biochar seems in many cases to effectively reduce Cr bioavailability through different immobilization mechanisms [212,213,214,215,216]. However, the opposite ionic nature of trivalent and hexavalent forms of Cr found in soils (cationic for Cr(III) and anionic for Cr(VI)), as well as the overriding toxicity concerns for Cr(VI), necessitates specialized application techniques, which include the prioritizing of Cr(VI) reduction at the more stable and less toxic Cr(III) form, and subsequently its immobilization processes via adsorption [214]. The above are highly dependent on pH and aerobic or anaerobic conditions found in soils or the amendments that are to be used, as well as the ionic charge that dominates upon their surfaces.

The transformation or degradation of Cr through biochar application includes redox reactions mainly involving the quinone and aromatic groups in its structure. Concerning the pH status, and unlike the adsorption behaviors of the cationic metals toward biochar, the removal capacity of Cr(VI) is significantly limited with increasing solution pH, whereas its highest removal capacity can be obtained under low pH conditions [139]. However, the presence of several fused rings of polycyclic aromatic hydrocarbons may serve as π-electron donor for Cr(VI) reduction to Cr(III), even at high pH ranges. Arshad et al. (2017), studying the effects of a combined application of biochar and bacteria on a sandy-loam soil to reduce Cr(VI) phytotoxicity and pyhtoavailability to wheat (*Triticum aestivum* L.) reported that a dosage of 5% biochar alone, despite the fact that it increased soil pH from 7.2 to 8.1, showed a relative high performance of reduction of Cr(VI) to Cr(III) (up to 48%) [217]. Nevertheless, the same authors reported that the combination of 5% biochar and chromium reducing bacteria (CRB) in consortia was found more effective, recording the highest reductive transformation of available Cr(VI) both in soil (99%), as well as in roots (98%) and shoots (97%).

In the same line, as far as the need of specific modification of biochars to enhance their Cr reduction properties is concerned, Xu et al. (2019) in a recent study, reported that the recorded inability of the biochar redox moieties to effectively interact with inorganic contaminants in many cases, might make the use of a redox mediator a useful tool in enhancing their immobilization properties [218]. Thus, they proposed the use of Fe as an electron shuttle between negatively charged biochar and Cr(VI) anions, showing that when Fe(III) was adsorbed onto the biochar surface, it was further reduced to Fe(II), resulting in the subsequent reduction of Cr(VI) into Cr(III). However, they reported that the enhancement effect of Fe(III) in Cr(VI) reduction was closely dependent on the acidity of the solution, Fe(III) concentration, as well as the temperature of biochar preparation.

### 5.5. Pb

Lead (Pb) is a widespread pollutant in the soil environment on a global scale with a long residence time, compared to other inorganic pollutants. It is not sensitive to redox reactions over the normal range of soil Eh and pH values and its geochemical behavior is dominated by the divalent cation Pb^2+^. Lead is a priority pollutant due to the potential hazard to human health via the consumption of edible species grown in Pb-contaminated soils and transferred to the food chain. Numerous studies have addressed the importance of different types of biochar at various pyrolysis temperatures as a very effective tool for the treatment of Pb-contaminated soils. 

Like other divalent metals, Pb showed similar mechanisms of interaction with biochars, e.g., surface adsorption, ion exchange, precipitation, and complexation and recent studies reported that compared to most PTEs, Pb is relatively easier to immobilize. The decrease of Pb availability in biochar-treated soil is likely due to the associated increase of soil pH, as reported by many studies. Such immobilization has been observed by Ahmad et al. (2017) in agricultural soils with alkaline pH conditions (>8.0), which was induced by biochars produced at 700 °C with high pH values of 9.7–10.4 [219]. Moreover, biochar’s high P concentration, like in animal manure-based biochars, could provide sorption sites to immobilize Pb via the formation of Pb_3_(PO_4_)_2_, a well-known mechanism to decrease the mobility of Pb in soil [220]. Furthermore, the cation exchange and precipitation reactions between inorganic components (CO_3_^2−^, OH^−^ and Ca^2+^, Mg^2+^) in biochar and Pb(II) are able to achieve a significant immobilization effect of Pb, such as the formation of Pb_3_(CO_3_)_2_(OH)_2_ precipitation. Ippolito et al., using lodgepole pine or tamarisk biochar in metal contaminated soils observed up to a 100% decrease in bioavailable Pb, as the result of precipitation process [221].

Lu et al. (2014) reported that the addition of 5% rice straw biochar to a sandy loam soil with pH~5.7 contaminated with Pb (527 mg kg^−1^) may have contributed to the 71% reduction in Pb concentration in the *Sedum plumbizincicola* shoots [110]. The application of cane straw-derived biochar (BC), produced at 700 °C to a metal contaminated mine soil, decreased the content of available Pb (DTPA extraction) reducing at the same time its uptake by *Canavalia ensiformis* and *Mucuna aterrima* [222].

Results showed that biochars made from cow manure and municipal compost at 300 and 600 °C treatments, significantly enhanced Pb immobilization attributed to their favorable chemical properties which could promote Pb conversion into stable chemical fractions [223]. Bian et al. (2014), reported that wheat straw biochar reduced Pb bioavailability by ~60% in paddy soils, reducing Pb rice uptake by 69%, which enhances food security [224]. Similarly, in a study conducted by Park et al. (2011), application of biochar derived from green waste to shooting range and spiked soils significantly immobilized and reduced the bioavailability of Pb *Brassica juncea* L. Czern. plants [225]. Qin et al. (2018), showed that the addition of pig manure biochar to Pb contaminated soil reduced the Pb bioavailable forms by 71% compared to untreated soils [226]. 

Ahmad et al. (2016) showed that the addition of soya bean stover and pine needle-derived biochars (produced at 300 and 700 °C) at application rate 10 wt% decreased the available Pb fractions in a polluted, alkaline, sandy loam soil up to 80% [227]. Similarly, Li et al. (2019) showed that the application of coconut fiber biochar (produced at 500 °C) applied at 2 and 4 wt% to a contaminated acid, loam soil decreased the exchangeable (33–40.8%) and carbonate (11.9–17.7%) Pb fractions [228]. Boostani et al. (2019) reported that the application of sheep manure and vermicompost biochars (produced at 300 and 500 °C) at 2 wt% in a contaminated soil resulted in a significant decrease in exchangeable (10.4–19.6%) and carbonate (3.8–10.5%) fractions of soil Pb [229]. Likewise, a significant reduction of Pb available fractions have been found by Salam et al. (2019) using rapeseed residue and rice straw biochars at 5 wt% in a contaminated, slightly acid soil [230]. Although field studies are fewer than the laboratory or greenhouse studies, their results suggested that, on average, 50% of Pb was immobilized by the application of biochar at rate > 10 t ha^−1^. 

### 5.6. Cd

Cadmium accumulation in vegetables is an issue of major environmental concern. Alleviated levels of Cd in the soil can affect plant growth in many ways, such as by eliminating yield, quality, root growth, and by inhibiting physiological procedures or causing structural changes in plants. Moreover, it has been well documented that when leafy vegetables are grown in Cd contaminated soils, they tend to accumulate high concentrations of Cd, posing a threat to human health. The review of Rizwan et al. (2017) refers to strategies that have been used for reducing Cd accumulation in plants [231]. Among them, biochar addition to soils has attracted the attention for the past decade, for its potential to immobilize PTEs such as cadmium. According to O’Connor et al. (2018), Cd is the most common pollutant in concern and has been explicitly investigated in numerous remediation in situ field trials [11]. In the majority of cases, experiments are conducted using soils derived from sites with high Cd levels due to geochemical reasons [111,232,233], contaminated soils due to human activity [110,174,175] or in pot experiments with artificially contaminated soils with Cd [122,234,235]. Hamid et al. (2019) suggests that biochar as an organic amendment immobilizes cadmium in soil, through processes such as adsorption, complexation and ion exchange [236].

In more recent studies, sugarcane-straw-derived biochar, (produced at 700 °C) succeeded in reducing Cd uptake by Jack bean and *Mucuna aterrima*, grown in a heavy-metal-contaminated mine soil (Puga et al., 2015). Soils amended with biochar (1.5, 3.0, and 5.0% *w*/*w*) showed decreased available concentrations of Cd (extracted with DTPA) and Cd toxicity symptoms in plants were alleviated, in comparison with the unamended treatments [178]. Similarly, the application of biochar ameliorated the harmful effects of Cd in spinach plants, grown under Cd stress [237]. Cotton stick derived biochar (0, 3, and 5%) was applied in a Cd-spiked soil (0, 25, 50, 75, and 100 mg Cd kg^−1^ soil). Spinach plants grown in unamended soils showed symptoms of Cd toxicity, such as decreased growth and oxidative stress. Biochar application, especially at 5% rate, inhibited toxicity symptoms in spinach and resulted in dry biomass increase, regardless the Cd level in soil. Moreover, Cd concentration in spinach shoots was reduced by 53, 36 and 31%, for the 25, 50 and 100 mg Cd kg^−1^ soil, respectively, which is an important outcome in terms of food safety.

Xu et al. (2018) examined the efficiency of two different biochars (peanut shell biochar (PBC) and wheat straw biochar (WBC)) to reduce Cd availability of a mine derived soil and accumulation of Cd to rice (*Oryza sativa* L.) [112]. Using the BCR sequential extraction method, they revealed that biochar induced the transformation of the acid-soluble fraction of Cd to oxidizable and residual fractions, making them less available for rice. Moreover, biochar treatments reduced the levels of MgCl_2_-extractable Cd, compared to untreated soil. Specifically, addition of 5% biochar led to reductions of 40.4–45.7% in the content of MgCl_2_-extractable Cd, whereas PBC more effectively immobilized Cd than WBC. Both biochars inhibited the uptake and accumulation of Cd in rice plants, since Cd concentrations in grains were reduced by 22.9 and 29.1% for the PBC and WBC addition, respectively. The BCR sequential extraction method was also used by Bashir et al. (2018), who used three types of biochar (rice straw (RSB), rice hull (RHB), and maize stover (MSB)) at rates 1.5 and 3% in order to evaluate their impact on Cd mobility [238]. Similarly with the previous authors, their results showed that biochar can change the geochemical distribution of Cd fractions in soil. The studies of Cui et al. (2019) and Sun et al. (2020) suggest same usefulness of biochar derived from wheat straw and conclude that biochar as a material can play a significant role in reducing Cd bioaccumulation and transfer through food chain, improving thus human health [239,240]. The most recent research of Tu et al. (2020) investigated the potential mechanisms of biochar and bacteria (metal tolerant strain of *Pseudomonas* sp. NT-2) loaded biochar on the stabilization of Cd a contaminated soil by a 75-day pot experiment [241]. The results confirmed the feasibility of the application of NT-2 loaded biochar inoculant to reduce the human health risks of Cd in contaminated alkaline soils.

### 5.7. As

Soil contamination by arsenic (As) poses a severe threat to human, plant, and animal life, therefore is considered as a priority-controlled toxic pollutant in soils. In the soil environment, As occurs in organic and inorganic forms, and in two distinct chemical species: (i) Arsenite [As(III)], as a hydroxyl species (H_3_AsO_3_, H_2_AsO_3_^−^), and (ii) arsenate [As(V)], as an oxyanion (H_2_AsO_4_^−^ or HAsO_4_^2−^). The former occurs in reduced/anaerobic soil conditions and the latter when oxidized/aerobic soil conditions prevail. Besides redox potential, As solubility in soils is highly dependent on the soil properties (pH, soil texture, clay mineralogy, the competing ions, organic matter), as well as the As minerals themselves [242]. Extremely high concentrations, such as 250,000 mg kg^−1^, have been reported in various soils causing serious concerns associated with food security and human health and, therefore, the remediation of these areas has become a global priority. 

**Figure 5:**
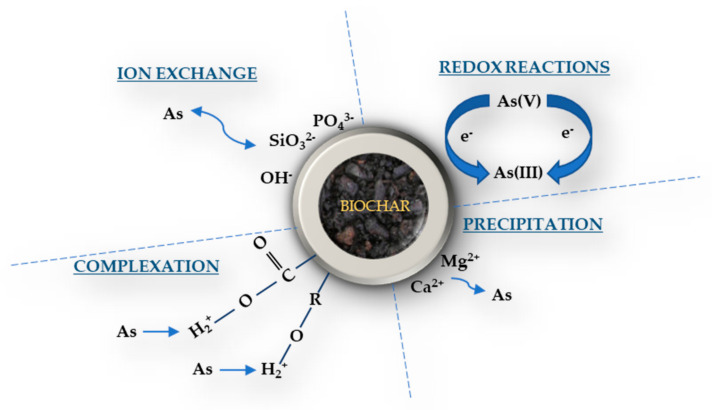
Potential mechanisms of biochar interactions with As in soil (modified from [134]).

Soil remediation technologies for As-contaminated soils, such as soil excavation, vitrification, landfilling, soil washing, and electrokinetic systems present increased cost and the danger of detrimental changes in the soil environment, therefore, biochar application can be considered as an alternative, environmentally friendly, cost-efficient, and more sustainable remediation technology. Whereas numerous studies have shown that biochar application had a positive impact on decreasing the bioavailability and bioaccumulation of As in soil-plant system, some researchers point out that biochar could enhance the accumulation of As in some plant species [243]. This contradiction supports the hypothesis that although the addition of biochar alters soil chemical properties, which enhance immobilization of cations (Cd, Pb, Ni), these changes are more favorable for mobilization of anions such as As. Due to the predominance of negative charges on most biochar surfaces, the sorption of As in the form of oxyanions onto biochar surfaces is highly restricted. This electrostatic repulsion, which leads to the decrease of As sorption by the soil components, is enhanced by the increase of soil pH due to biochar addition, as already supported by many researchers. Moreover, a rapid transformation of As(V) to As(III), due to the reducing ability of the surface-active functional groups of the biochar, has been observed in many biochars-As contaminated soil resulting in the increase of As(III) mobility. The literature highlights that the effect of biochar on the dynamics of soil As (Figure 5) was influenced by critical factors, including soil types, experimental conditions, biochar characteristics, as well as soil properties. 

Many studies have pointed out that the application of biochar in acid paddy soils increased the bioavailability of As and consequently the bioaccumulation of As in rice grain. By applying a rice husk biochar (RHB) in a heavily contaminated soil at various rates, Ibrahim et al. (2016) confirmed the increase of As accumulation in shoots, leaves, and roots of alfalfa (*Medicago sativa* L.) through increasing the available concentration of Si and P in soil [244]. Therefore, they pointed out that in a multi-metal contaminated soil, biochar addition may promote the mobility and bioavailability of As increasing the environmental hazard. Dixit et al (2003) concluded that the increase in the application rate of biochar from 30 to 60% in soil with ryegrass resulted in the increase of As in shoots of ryegrass. In this context, in order to improve the remediation efficiency of the biochar, many researchers have modified the original biochar with various amendments, such as Fe oxides, which show a great affinity for As, zero-valent Fe, Mn oxides and clay minerals in order to create novel biochars with enhanced sorption ability to As immobilization. Wu et al. (2018) applied iron-modified biochars with Fe-oxyhydroxy sulfate (Biochar-FeOS), FeCl_3_ (Biochar-FeCl_3_), and zero-valent iron (Biochar-Fe) at a rate of 1% in As-contaminated paddy soil which reduced the bioavailability of As by 13.95–30.35%, 10.97–28.39%, and 17.98–35.18%, respectively [91]. 

The same trend was observed when Li et al. (2018) added 4% modified biochar (MBC), 0.5% Fe grit as zero-valent iron (ZVI), 0.5% Fe grit + 4%MBC (ZMBC), 0.5% ZVI + 4% biochar (ZBC), 4% biochar (BC) in a contaminated As-soil (As 95.6 mg Kg^−1^). MBC and ZMBC were shown the more effective modified biochars decreasing the accumulation of As in the shoots and roots of *Brassica campestris* L. by 44.55% and 45.40% for MBC and 74.92% and 71.80%, for ZMBC, respectively [245]. In some cases, Fe oxides can be present naturally in biochars in various amounts depending on the type of feedstocks and pyrolysis conditions. These oxide minerals can control the bioavailability of As forming inner-sphere complexation especially when available P is low in the soil. 

Regarding the soil microbial activity, it increases when biochar is applied in soil via the increase of DOC which, in turn, increases the mobility of As in soil because it enhances the microbial reduction of As(V) to As(III) [244]. However, the soil-microbial interactions in As contaminated soils, after the addition of biochar, are quite complicated and since only few studies have been carried out, more research is necessary on this topic. 

## 6. Future Perspectives and Conclusions

With this review, we highlighted the steady growth on biochar research in recent years, a fact which indicates its potential value as an efficient alternative in PTEs immobilization in contaminated soils. Using the CiteSpace visual scientometric analysis tool, our study showed that between years of 2019 and 2020, research sub-fields like earthworm activities and responses, greenhouse gases emission, and low-molecular-weight organic acids have gained most of the attention when biochar was investigated for soil remediation purposes. Moreover, biomass like rice straw, sewage sludge, and sawdust were found to be the most commonly used feedstocks for biochar production. In addition, and as seen from our literature review on the use of biochar on contaminated soils studies, the interaction between biochar and PTEs is usually governed by processes such as electrostatic attraction, ion exchange, complexation, and precipitation. The above immobilization mechanisms strongly depend on potential interactions of biochar on existing soil chemistry and are highly governed by specific soil chemical properties like pH, EC, CEC, or soil nutrient status. 

**Figure 6:**
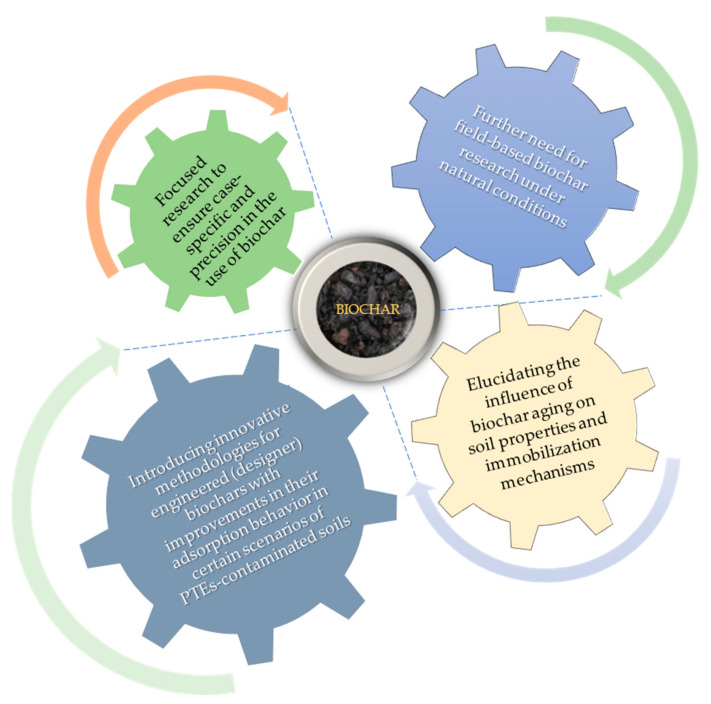
Future perspectives for a more focused soil remediation strategy with biochar application.

However, although many studies have been conducted regarding biochar application on contaminated soils with PTEs, very few of these studies have been field-based (Figure 6). We consider this to be a major research gap, since specific natural conditions like temperature, rainfall, wind, pH, etc. may significantly influence the sorption capacity and consequently the immobilization mechanisms of biochars. Thus, more studies focused on biochar application in the field under natural conditions are required to fully understand and elucidate the above mechanisms (or their coordination in terms of their independent or potentially complementary action) and, additionally, to ensure that biochar application will be economically feasible and/or practical in terms of obtaining the initial biomass for biochar production. 

Given biochar’s general high resistance to biological decay, as well as the fact that its stability in the soil environment is variable and highly site-specific, biochar aging may strongly alter its physicochemical and biological properties. Though the effect of fresh biochar on soil characteristics attracted the attention of the research community, the influence of biochar aging effects on soil properties and immobilization mechanisms has been the subject of comparatively fewer scientific attempts.

Moreover, the fact that results being reported in literature concerning biochar ap-plication on polluted soils with PTEs might emerge from experiments conducted under different conditions e.g., pyrolysis heating rates, reactor capacities, inert gas atmospheres, temperatures, and residence times, constitutes an additional issue of lack of uniformity. The latter makes any effort to correlate groups of biomasses to biochar properties rather difficult, and therefore significant work is required to ensure use of biochars is case-specific and done with precision. The same also stands with respect of factors such as biochar type, application rate, as well as application time. 

Finally, as more extensively discussed on Section 2.3, introducing innovative methodologies for engineered (designer) biochars with improvements in their adsorption behavior in certain scenarios of PTEs-contaminated soils is one of the main research challenges for producing more efficient biochars. Thus, emerging biochar engineering strategies like deposition of zero valent iron nanoparticles (nZVI) on biochar, single-stage production of hydroxyapatite/biochar composites, and biochar loading with bacteria efficient in adsorbing PTEs like Cr, are some of the many promising efforts that form part of a focused remediation strategy.

## Figures and Tables

**Table 1:** The list of 21 inorganic elements that are commonly regarded as PTEs in environmental studies [1].

Transition Metals:	Ag,	Cd ^1^,	Co,	Cr ^1^,	Cu ^1^,	Fe,	Hg,	Mn,	Mo,	Ni ^1^,	V,	Zn ^1^
Post-transition metals:	Al,	Pb ^1^,	Tl									
Metalloids:	As ^1^,	B,	Sb									
Alkali metals:	Ba,	Be										
Non-metals:	Se											

^1^ Seven PTEs, including Cd, Cr, Cu, Ni, Zn, Pb, and As are considered the most common elements found in contaminated soils and are analyzed in this paper. For elements like Se, knowledge gaps exist, therefore more systematic studies should be conducted to investigate the effect of biochar on their availability.

**Table 2:** Network modularity, cluster silhouette, the number of papers and the most frequently reported terms in the top 6 clusters in 2019 and 2020.

2019 (Network Modularity: 0.633)			
Cluster #	Silhouette	Number of Papers (Out of Year Total)	Most Frequently Reported Common Terms among the Papers of Each Cluster
0	0.675	77	Biochar, effect, growth, radish, Pb accumulation, heavy-metal contaminated farmland
1	0.863	49	Biochar, rapid removal, triazine pesticides, adsorption mechanism, chlorpyrifos-methyl adsorption, biochar synthesis
2	0.932	42	Biochar, multi-analytical characterization, agricultural applications, waste biomasses, hydrochar, composting
3	0.869	37	Biochar, soil, availability, compost, 4-tetrabrominated diphenyl ether, plant uptake
4	0.861	37	Soil, polycyclic aromatic hydrocarbons, oxalic acid, maize straw biochar, mechanistic study, dissipation
5	0.888	36	Biochar, effects, cadmium accumulation, rice grains, pollution level, tungsten-mining area field experiment
6	0.894	29	Biochar, soil, pesticide mesotrione, eisenia, geochemical fractions, phytoavailability
**2020 (Network Modularity: 0.608)**			
0	0.799	61	Biochar, water, irrigation use, graphene oxide, magnetic biochar composites, cadmium adsorption
1	0.798	49	Biochar, remediation, heavy metals, calcium silicate hydrate, growth performance, amendment
2	0.723	46	Biochar, cadmium, brassica napus, Enterobacter sp., phytotoxic impacts, oxidative disturbances
3	0.760	39	Biochar, polluted soil, enzymatic activity, bacteria, biochar enhanced composite, Cu immobilization
4	0.766	38	Biochar, soil, accumulation, transformation, zea mays, heavy metals
5	0.716	37	Biochar, accumulation, heavy metals, zea mays, transformation, processed fly-ash
6	0.856	34	Biochar, phytoremediation, safe use, treatment, biomass, oil crop rotation
